# Ecological Drivers of the Soil Microbial Diversity and Composition in Primary Old-Growth Forest and Secondary Woodland in a Subtropical Evergreen Broad-Leaved Forest Biome in the Ailao Mountains, China

**DOI:** 10.3389/fmicb.2022.908257

**Published:** 2022-06-13

**Authors:** Qingchao Zeng, Annie Lebreton, Xiaowu Man, Liukun Jia, Gengshen Wang, Sai Gong, Marc Buée, Gang Wu, Yucheng Dai, Zhuliang Yang, Francis M. Martin

**Affiliations:** ^1^Beijing Advanced Innovation Center for Tree Breeding by Molecular Design, Beijing Forestry University, Beijing, China; ^2^School of Ecology and Nature Conservation, Beijing Forestry University, Beijing, China; ^3^INRAE, UMR Interactions Arbres/Microorganismes, Centre INRAE-GrandEst-Nancy, Université de Lorraine, Champenoux, France; ^4^Chinese Academy of Sciences Key Laboratory for Plant Diversity and Biogeography of East Asia, Kunming Institute of Botany, Kunming, China; ^5^Yunnan Key Laboratory for Fungal Diversity and Green Development, Kunming, China

**Keywords:** community structure, evergreen sclerophyllous broad-leaved forest, forest replacement, pine forest, subtropical biome, soil microbiome, Yunnan

## Abstract

Replacement of primary old-growth forests by secondary woodlands in threatened subtropical biomes drives important changes at the level of the overstory, understory and forest floor, but the impact on belowground microbial biodiversity is yet poorly documented. In the present study, we surveyed by metabarcoding sequencing, the diversity and composition of soil bacteria and fungi in the old-growth forest, dominated by stone oaks (*Lithocarpus* spp.) and in the secondary Yunnan pine woodland of an iconic site for biodiversity research, the Ailaoshan National Nature Reserve (Ailao Mountains, Yunnan province, China). We assessed the effect of forest replacement and other environmental factors, including soil horizons, soil physicochemical characteristics and seasonality (monsoon vs. dry seasons). We showed that tree composition and variation in soil properties were major drivers for both bacterial and fungal communities, with a significant influence from seasonality. Ectomycorrhizal Operational Taxonomic Units (OTUs) dominated the functional fungal guilds. Species richness and diversity of the bacterial and fungal communities were higher in the pine woodland compared to the primary *Lithocarpus* forest, although prominent OTUs were different. The slightly lower complexity of the microbiome in the primary forest stands likely resulted from environmental filtering under relatively stable conditions over centuries, when compared to the secondary pine woodlands. In the old-growth forest, we found a higher number of species, but that communities were homogeneously distributed, whereas in the pine woodlands, there is a slightly lower number of species present but the communities are heterogeneously distributed. The present surveys of the bacterial and fungal diversity will serve as references in future studies aiming to assess the impact of the climate change on soil microbial diversity in both old-growth forests and secondary woodlands in Ailaoshan.

## Introduction

In natural and managed forest ecosystems, soil microbial communities drive ecological processes, such as soil geochemical cycles, carbon sequestration, nutrient cycling, and productivity (Clemmensen et al., 2013; Tedersoo et al., 2015; Bahram et al., 2018). They also shape the plant phenotypes, including their functional traits, growth, survival, reproduction, ecological strategies, nutrient use, and stress responses (Wardle et al., 2004). Moreover, plant-microbial interactions, such as mycorrhizal symbiosis, are important determinants of plant biogeographic range sizes, population dynamics, and community composition (Van der Heijden et al., 2015; Wagner et al., 2016; Tedersoo et al., 2020). Microbial communities are intermingled through trophic networks that rapidly respond to changes in environmental conditions, such as nutrient levels, precipitations or droughts. Therefore, a complete understanding of forest ecosystem functioning relies on a comprehensive understanding of the environmental factors driving the structure of microbial communities growing in association with tree roots.

High-throughput metabarcoding sequencing has facilitated large scale research studies describing the extent of microbial richness in hundreds of terrestrial biomes (Zimmerman and Vitousek, 2012; Hartmann et al., 2014; Tedersoo et al., 2014, 2015, 2020). These surveys have unraveled high levels of soil microbial diversity, much of it resulting from the presence of unknown species. Despite of these new monitoring tools and a growing appreciation for the diversity of the soil microbiome, it has proved to be rather challenging to decipher the spatial and temporal patterns in bacterial and fungal diversity and their drivers in tropical and subtropical forests, often located in remote regions with rough topography unsuitable to agriculture (Wardle et al., 2011; Peay et al., 2013). Throughout the world, mountain biomes are experiencing major climate warming, forest replacement and deforestation (Hagedorn et al., 2019). Although responses of subtropical montane forests to global changes have been documented aboveground, there are also alterations taking place belowground, where plant roots and their associated microbial communities establish complex, but largely unknown entangled networks in the soil (Toju et al., 2014; Wu et al., 2014; Adamo et al., 2021). To our knowledge, limited information exists on the adaptation of microbial communities, such as modification of their distribution pattern, life history and functional traits, to forest shift in subtropical montane forests (Miyamoto et al., 2015; Geml et al., 2017).

Therefore, assessing soil microbial diversity in endangered subtropical old-growth forests is required as several keystone species that are important to these ecosystems may disappear before they are even discovered. The extent to which tree species and soil horizons affect quantitative and qualitative traits of the belowground microbial communities in subtropical forests should also be investigated. To determine the possible effects of forest replacement on the soil microbiome, we compared several stands in a primary old-growth broad-leaved evergreen cloud forest, dominated by stone oaks (*Lithocarpus*) species, and secondary native pine (*Pinus yunnanensis*) woodlands in a ‘hot spot’ of biodiversity, the Ailaoshan National Nature Reserve (NNR) located on the northern crest of the Ailao Mountains (Ailaoshan), in central-west Yunnan. This evergreen sclerophyllous broad-leaved forest biome is the most extensive subgroup in the subtropical evergreen broad-leaved forest which historically covered most of southern China (Young et al., 1992). We sought to acquire key information on the diversity of soil bacteria and fungi between adjacent contrasting sites that differ in human impact and as a consequence, the dominant tree species (stone oaks vs. Yunnan pines), across a landscape encompassing similar environmental gradients. The Ailaoshan NNR resides in the center of the largest subtropical land area in the world (Young and Wang, 1989). This region is a major climatic border between China’s southwestern and southeastern monsoon systems, and the northern Himalaya Plateau and is recognized for its high plant biodiversity. Unfortunately, the remaining primary forests currently face tremendous pressures because of recent increases in population and human activities. By 2020, the forest cover had dropped to 30% and continues to decline^1^. When the original broad-leaved evergreen cloud forest is destroyed, the native Yunnan pine (*Pinus yunnanensis*) and Simao pine (*Pinus kesiya*) are generally the first secondary tree species to spontaneously invade the site. Primary forest replacement by secondary woodlands drives important changes at the level of the overstory, understory and forest floor, including plant and litter transformation, microclimatic conditions and edaphic properties, but the effects on belowground microbial diversity and the ecosystem functions it provides are poorly understood.

The aims of present study were to characterize the bacterial and fungal communities in an evergreen sclerophyllous broad-leaved subtropical forest and its secondary pine woodland replacement, including taxonomic composition, to detect potential shifts in richness, functional guilds, and community composition and to assess the roles of environmental drivers. We thus tackled the following questions: How does spatial and temporal change in soil and root microbial diversity among forest sites reflect major environmental features, such as soil physicochemical characteristics and seasonality? Do tree individuals in the primary evergreen broad-leaved forest host the highest microbial diversity compared to the secondary pine woodlands? To answer these questions, we have used culture-independent, high-throughput ribosomal DNA amplicon sequencing to investigate variation patterns in soil and root microbial communities. Surveying the structure of both bacterial and fungal communities and their associated functions can improve our ability (1) to survey changes of the soil system after replacement disturbances, (2) to assess its ability to regenerate, and (3) to identify adverse alterations in biogeochemical cycles before they are irreversible.

## Materials and Methods

### Study Sites

The study area is located in the Ailaoshan Subtropical Forest Ecosystem Research Station (24°31′ N, 101°01′ E; referred as to the Ailaoshan Station) in the northern section of the Ailaoshan Nature Reserve^2^ (Xujiaba region, Jingdong County, Yunnan Province, China). The Ailaoshan lie between the southern and northern subtropical forest formations in a transition area. The most extensive forest ecosystem is a contiguous primary, old-growth broadleaf evergreen stone oaks (*Lithocarpus*) association which covers 75–80% of the region (Young et al., 1992).

**FIGURE 1:**
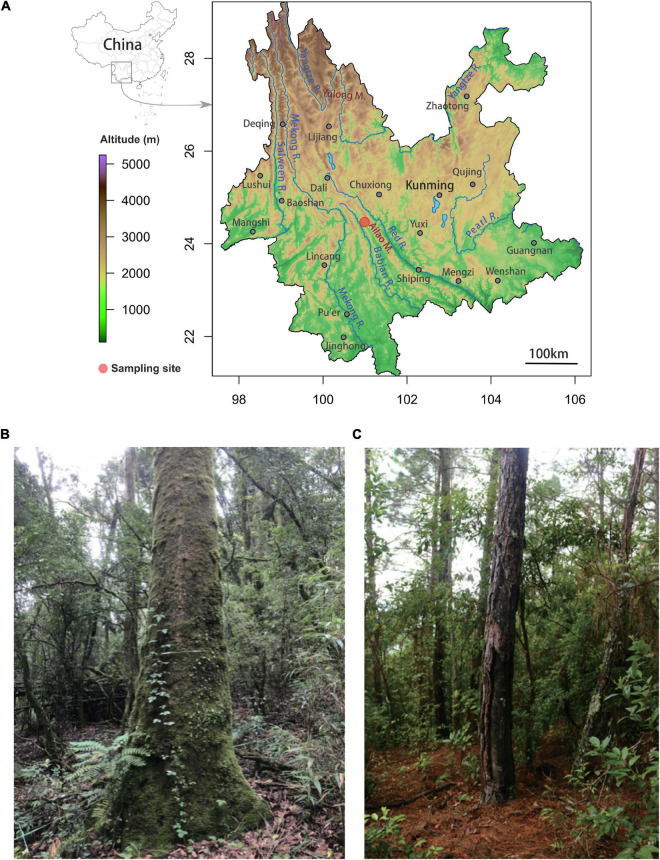
Location of the primary old-growth stone oak (*Lithocarpus*) forest and the secondary *Pinus* plantation in the Ailaoshan National Nature Reserve at Xujiaba Region. **(A)** The upper map shows the location of the reserve (red dot) in Central Yunnan. The photos illustrate the different forest associations where the soil cores were sampled with **(B)** the native, primary old-growth *Lithocarpus* forest and **(C)** the *Pinus yunnanensis* plantation.

The surveyed old-growth primary forest lies at 2500 m elevation in flat areas within a protected section of 5100 ha of evergreen forest with a stand age of more than 300 years, free of management (Yang et al., 2007; Song et al., 2017; Figure 1 and Supplementary Figure 1). The tree density of the forest is 2728 stems per ha^–1^ (Wen et al., 2017). The dominant tree species at the Ailaoshan Station hail from the Fagaceae, Theaceae, and Lauraceae families. In total, there are 104 species represented in 44,168 stems, including the dominant canopy trees *Lithocarpus chingdongensis*, *Lithocarpus xylocarpus*, *Lithocarpus hancei*, *Castanopsis wattii*, and *Schima noronhae* (Schaefer et al., 2008; Wen et al., 2017). The upper canopy of the forest is 18–25 m high. Diameter at breast height (DBH) of the selected stone oaks ranged from 75 to 180 cm. The soils are loamy Alfisols (Chan et al., 2006; Wen et al., 2017). At this site, the net primary production (NPP) is ∼10 tC ha^–1^ yr^–1^ (biomass increment plus litterfall production), the annual sum of net ecosystem production (NEP) is ∼9 tC ha^–1^ and the mean annual carbon sequestration rate by biomass and necromass is ∼6 tC ha^–1^, while the annual litterfall production is 4.3 tC ha^–1^ yr^–1^ (Tan et al., 2011). The total microbial biomass in the humus layer (4.33 ± 1.64 g C kg^–1^ dried soil) was approximately double that in the mineral soil (2.16 ± 0.88 g C kg^–1^ dried soil) (Chan et al., 2006).

Meteorological data were obtained from the Ailaoshan meteorological station located a few km away from the study sites. Available climate variables include precipitation, annual mean air temperature at 2 m height and soil temperature at 10 cm depth, relative humidity, and photosynthetic active radiation. Influenced by the southwest and southeast monsoons, the climate of the Ailaoshan alternates between wet and dry conditions. Mean annual precipitation is 1799 mm, 86% of which occurs during the monsoon rainy season from May to October (Song et al., 2017). The annual mean temperature of 11.6°C, with monthly mean values ranging from 5.4 to 23.5°C (1982–2015). Daily climate conditions from the Ailao meteorological station for the period of January 2012 to December 2017 can be found in Fan et al. (2019). Seasonal variability in soil temperature and soil water content can also be found in Wu et al. (2014).

Outside of the Ailaoshan NNR, the mountain sides have been virtually stripped of their original vegetation leaving croplands, pastures and woodlands of native Yunnan and Simao pines. The Yunnan pine woods (2050 m, Figure 1 and Supplementary Figure 1) where we conducted the soil samplings was an open, park-like forest with a minimum of human interference, although several older trees were resin-tapped. The sampling locations were roughly flat except for the area of cluster 2 which situated on a slope. The areas studied were once covered by evergreen broadleaf forest about 55–65 years ago (Young and Wang, 1989). In pine woods, *P. yunnanensis* dominated a mix of broadleaved evergreen species such as *Alnus nepalensis*, *Gaultheria forrestii*, *Lyonia ovalifolia*. The DBH of the selected pine trees ranged from 65 to 95 cm. These two forest associations present very few similarities and share a restricted set of plant species, even though they grow a few kilometers apart and the Yunnan pine woodland developed where the stone oak old-growth forest once stood.

Because of logistical constraints, vegetation data were not collected during sampling. However, the distribution of the site vegetation, including tree community data, has been carried by Young and Wang (1989); Young et al. (1992), Yang et al. (2007), and Wen et al. (2017). The sampled plots in the old-growth forest are dominated by the ectomycorrhizal hosts *Lithocarpus hancei* and *L. xylocarpus* (stone oaks) (57% of the relative basal area) and *Castanopsis wattii* (22% of the relative basal area), while the pine woodland is mainly comprised of the ectomycorrhizal *P. yunnanensis*.

At each site, we contemporaneously surveyed the bacterial and fungal communities of soil and root samples associated with mature, healthy trees. We designed the sampling sites comprising three plots each with four trees, verified that sampling was sufficient for inter-site comparisons (Supplementary Figure 1), and compared richness between sites, soil layers and seasons.

### Soil Sampling

Soil cores were collected during the wet season on early August 2019, at the end of the dry season on April 2020, and during the wet season on early August 2020. Six plots were sampled, three beneath *Lithocarpus* in the old-growth forest and three beneath *P. yunnanensis* in the pine woodland. Plots were separated by ∼100 m. Within each plot, we sampled four individual trees separated by 5 to 15 m (see Supplementary Figure 1 for maps) to ensure sample independence. At each sampling location, we collected soil cores to survey the fungal and bacterial communities, and soil physicochemical characteristics. Two soil cores were collected at 1 m (north- and southward) from the base of the trunk. Litter or plant detritus were discarded. The humic fragmented (HF; thickness of 2–5 cm) soil horizon, i.e., forest floor, was collected and the underneath soil cores were then subsampled in organic soil (OS; depth of 0–5 cm) and organo-mineral soil (OM; depth of 5–25 cm). Soil samples were immediately sieved through 5-mm meshes, homogenized, subsampled in sterile plastic tubes and snap frozen in dry ice. This spatially explicit sampling scheme totaled 144 fungal community samples (2 sites × 3 plots × 4 trees × 2 soil cores × 3 soil horizons) and 144 bacterial community samples per foray. Four soil cores were of poor quality and were discarded. Samples were kept in dry ice chests until back to the Kunming Institute of Botany (KIB) where samples were then stored at –80°C until further DNA extractions and physicochemical soil analyses.

We sampled roots found in the soil cores beneath the stone oaks or Yunnan pines at 2 m from the base of the trunk at the 144 sampling positions in April and August 2020. At each sampling position, we harvested oak or pine root fragments (approximately 2 cm long), washed them with distilled water and immediately stored them on dry ice until back to KIB where samples were then stored at –80°C until further DNA extraction. All necessary permits for the sample collection were issued by the Ailaoshan Station.

### Physicochemical Soil Analyses

Physicochemical characteristics were measured following protocols detailed by Bao (2000). It includes pH, total nitrogen (N), phosphorus (P), calcium (Ca), soil organic matter (SOM), and cation exchange capacity (CEC). Analyses were carried out by Puluo Environmental Protection Technology Co., Ltd (Guangzhou, China).

### DNA Extraction, Amplification and Sequencing

Total DNA was extracted from 50 mg of soil samples using the Qiagen DNeasy PowerSoil DNA Isolation Kit (Qiagen, Germany) according to the manufacturer’s protocol. Root samples were extracted using the cetyltrimethylammonium bromide (CTAB) procedure (Maropola et al., 2015). DNA quality and quantity were assayed using a NanoDrop ONE spectrophotometer (Thermo Fisher Scientific, Waltham, MA, United States). DNA extractions were carried out in triplicates and PCR products were pooled to reduce extraction-related variance. Amplicon libraries were prepared in a single PCR reaction using Illumina-barcoded fungal- or bacterial-specific primers. For identifying fungal Operational Taxonomic Units (OTUs), the primers ITS1F and ITS2 were used to target the hypervariable nuclear ribosomal ITS1 region (Gardes and Bruns, 1993), while the bacterial community was profiled by using the 16S rRNA V4 gene primers 515F and 806R, with barcodes at the 5′end of the forward primer (Caporaso et al., 2011). All PCR reactions were carried out in 30 μL reactions with 15 μL of Phusion high-fidelity PCR Master Mix (New England Biolabs, Ipswich, MA, United States), 0.2 μM of forward and reverse primers, and about 10 ng template DNA. The PCR amplifications were carried out using the following program: 1 min initial denaturation at 98°C, 30 cycles of 10 s at 98°C, 30 s at 50°C, and 30 s at 72°C, with a final 5 min elongation at 72°C. Both a negative control (ddH_2_O with no DNA template) and a positive control sample (an artificial DNA molecule with multiple primer sites) were used to assess obvious contamination during sample preparation for PCR and the efficiency of PCR, respectively. Libraries were generated using Illumina TruSeq DNA PCR-Free Library Preparation Kit (Illumina, United States) following the manufacturer’s recommendations and index codes were added. The library quality was assessed on the Qubit 2.0 Fluorometer (Thermo Fisher Scientific, Waltham, MA, United States) and Agilent Bioanalyzer 2100 system. Finally, the library was sequenced on an Illumina NovaSeq platform at Novogene Biotech (Beijing, China) and 250 bp paired-end reads were generated.

### Processing and Analysis of the Sequencing Data

Amplicons were analyzed using VSEARCH (v2.13.3) and QIIME (v1.9.1) (Caporaso et al., 2010; Zgadzaj et al., 2016). Raw sequences were split according to their unique barcodes, and adaptor and primer sequences were trimmed by the sequencing company. Then, we merged paired-end sequences by using the USEARCH software (v11.0.667). Output sequences were then quality-filtered (fastq_maxee_rate 0.01 -fastq_maxns 0) and singletons were removed using VSEARCH. Reads were clustered into OTUs at 97% similarity level using the UPARSE pipeline. Chimeric sequences that were identified using the reference-based chimera checking methods were removed from the data. Sequences were queried using the BLAST algorithm against the SILVA v132 ribosomal RNA (rRNA) database (Quast et al., 2012) and UNITE v.8.2 database (Nilsson et al., 2019) for fungal rDNA sequences in QIIME. Mitochondrial and chloroplastic DNA sequences, as well as OTUs with a total relative abundance < 0.00001 in all of the samples were discarded. We produced a total of 35,668,160 and 32,620,949 high-quality sequences for bacterial and fungal amplicons, which were clustered into 7,660 and 2,507 OTUs (> 97% sequence similarity) for bacterial and fungal OTUs, respectively. In order to predict fungal functional properties, ecological guilds (e.g., ectomycorrhizal fungi, pathogenic fungi and saprotrophic fungi) were attributed according to the FungalTrait database (Põlme et al., 2020), the mapping was performed at genus scale. Raw sequences have been submitted to the Sequence Read Archive under accession number PRJNA782391.

To assess the impact of data processing, we also analyzed these sequences using FROGS v3.1.0 (Escudié et al., 2018) with similar results (data not shown). In addition to these OTU workflows, the alternative, widely used amplicon sequence variant (ASV) approach (Callahan et al., 2016) was carried out using DADA2 (v1.18). The patterns of community composition and richness based on OTUs and ASVs showed strong correlations (data not shown). We will only discuss the results based on OTUs as the ASV approach tends to eliminate taxa that are both rare and phylogenetically unique (Joos et al., 2020).

### Statistical Analysis

To assess the microbial alpha-diversity, Shannon, chao and observed richness indices were calculated with QIIME 1.9.1. Beta-diversity was estimated according to the Weighted UniFrac distance between the samples. The OTU tables were rarefied to the lowest number of reads for alpha-diversity and used as a normalization method for beta-diversity analysis. We used non-metric multidimensional scaling (NMDS) (with Weighted UniFrac dissimilarity) to visualize community structure and permutational analysis of variance (PERMANOVA) as implemented in the Vegan R package (Oksanen et al., 2007) to quantify variation. The PERMANOVA (adonis) was iterated with 1,000 permutations. A linear mixed model was used to identify the major drivers of bacterial and fungal alpha-diversity (Nakagawa et al., 2013). The model was set using the dominant tree species, the season and the soil layer as fixed effects and the alpha diversity indices as independent variables [Div.fit1 = lmer (shannon/chao1 ∼ Tree species*Season * Soil layer + (1| block),REML = TRUE, data = sp)].

For the differential abundance analysis, we used STAMP (Parks et al., 2014). Welch’s tests were performed at phylum and class levels between *Lithocarpus* and *Pinus* samples. To identify the microbial taxa responsible for the community differentiation among forest association, we used a generalized linear model (GLM) approach implemented in *EdgeR* to identify microbial taxa with significant abundance differences among forests (Robinson et al., 2010). The differential OTUs with false discovery rate-corrected *P*-values < 0.05 were identified as indicator OTUs. While, it illustrated by ternary plots with the *ggtern* package (Hamilton and Ferry, 2018) for each sampling season or soil layer. In this study, we defined dominant taxa as described by Xiong et al. (2021a) associated, on one side to *Lithocarpus* forest samples and on the other side to *Pinus* forest samples. In these two subsets, OTUs present in at least 80% of the samples for the bacterial community or 50% of samples for the fungal community and having a relative abundance ≥ 0.1% of the subset reads were considered as dominant. The Venn diagram was drawn using the OECloud tools^3^ to identify shared and specific OTUs. Phylogenetic tree was annotated and visualized in iTOL software^4^ (Letunic and Bork, 2019). Linear discriminant analysis effect size (LEfSe) was applied (Wilcoxon *p*-value < 0.05, logarithmic LDA score > 2^5^) to identify the biomarker for each forest association and sampling season. Spearman’s correlation analysis was used to detect the relationships between environmental variables and the microbial alpha-diversity. The Kruskal–Wallis test was used to evaluate the alpha-diversity and the taxonomical difference observed in different soil layer and different sampling season and the Wilcoxon rank sum test was used to evaluate the alpha-diversity of forest association. All statistical tests performed in this study were considered significant at *P* < 0.05.

## Results

### Soil Physicochemical Characteristics

**FIGURE 2:**
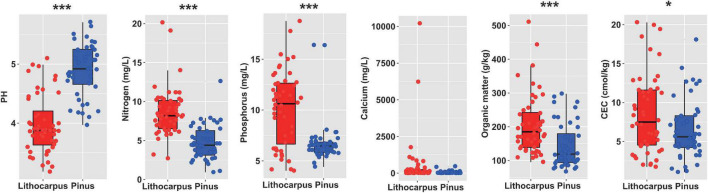
Physicochemical properties of the soil cores collected in the primary *Lithocarpus* forest and the secondary Yunnan pine plantations. The asterisks indicate the properties significantly different between *Lithocarpus* and *Pinus* samples (*P < 0.05; **P < 0.01; ***P < 0.001; one-way ANOVA). CEC, cation exchange capacity.

The N, P, SOM, and CEC content were significantly higher in the stone oak forest soils by comparison to the pine woodland soils. In contrast, pH was significantly higher in pine woodland soils (Figure 2). On the other hand, there was no significant effect on the other measured soil characteristics (ANOVA, *P* < 0.05, Figure 2). As expected N and SOM were significantly higher in the organic soil layer (OS) than the organo-mineral layer (OM). No significant effect of the soil layer was observed on the other measured soil characteristics (Supplementary Figure 2).

### Are Fungal and Bacterial Communities Different Between Forest Types?

**FIGURE 3:**
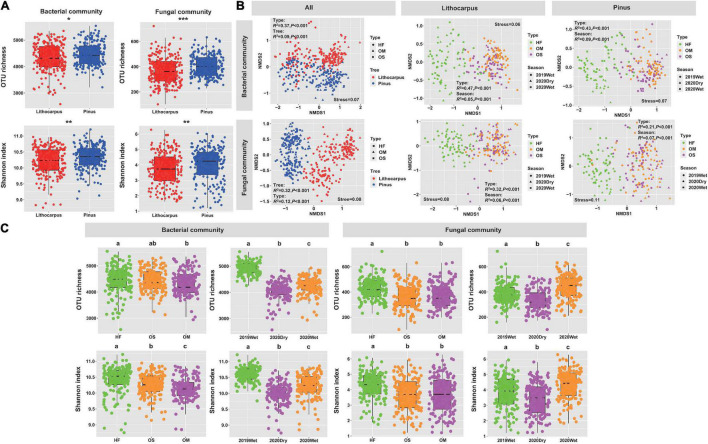
**(A)** Effects of the tree species shift from primary *Lithocarpus* association to secondary pine forest on soil bacterial and fungal richness and diversity. **(B)** NMDS ordinations based on UniFrac distance matrices of bacterial and fungal community across all samples, *Lithocarpus* and *Pinus* samples. **(C)** Diversity and richness of the bacterial and fungal communities in the soil layers and among seasons (*P* < 0.05; Kruskal–Wallis test). Asterisk in the different panels indicates significant differences between the forest sites (*P < 0.05; **P < 0.01; ***P < 0.001, Wilcoxon rank sum test). HF, Humic fragmented. OS, Organic soil. OM, Organo-mineral soil. 2019Wet, wet season 2019. 2020Wet, wet season 2020. 2020Dry, dry season 2020.

The alpha-diversity of soil bacterial and fungal communities was assessed by Shannon diversity and observed OTU indexes (Figure 3). Differences in bacterial and fungal richness were detected between the primary old-growth oak forest and late-successional secondary pine woodland. The alpha-diversity was slightly, but significantly, higher for both the bacterial and fungal microbiomes in the secondary Yunnan pine woodland by comparison to the primary stone oak forest (*P* < 0.05, Figure 3A). Ordination of soil microbial communities revealed a robust clustering of bacterial and fungal communities by soil layer and forest associations, with strong signatures of seasonality (dry vs. wet season) (Figure 3 and Supplementary Table 1) in defining soil bacterial and fungal microbiomes.

Within each site, the variation in the beta-diversity of bacterial and fungal communities was mainly related to the soil layer and seasonality (Figure 3B). Similar community patterns were observed during the wet and dry seasons (Figure 3C), although both the bacterial and fungal diversities were lower (*P* < 0.05) at the end of the dry season (i.e., 2020Dry) by comparison to the wet season. In both forests, we found that the soil cores collected during the monsoon in August 2019 displayed the highest diversity for the bacterial microbiome (Figure 3C), while this diversity and richness were the highest during the wet season 2020 for the fungal microbiome (Figure 3C). Furthermore, the linear mixed model analysis based on all samples showed that the diversity and richness of both bacterial and fungal communities were mainly influenced by seasonality (*F*_2_,_400_ = 432.72, *P* < 2.2e-16; *F*_2_,_400_ = 175.21, *P* < 2.2e-16; *F*_2_,_400_ = 27.98, *P* = 4.23e-12; *F*_2_,_400_ = 32.24, *P* = 1.0e-13) (Supplementary Table 2). The Spearman correlation analysis showed that pH and P content influenced the alpha-diversity of bacterial and fungal communities, respectively (Supplementary Table 3).

### OTU Distributions and Co-occurrence Patterns

**FIGURE 4:**
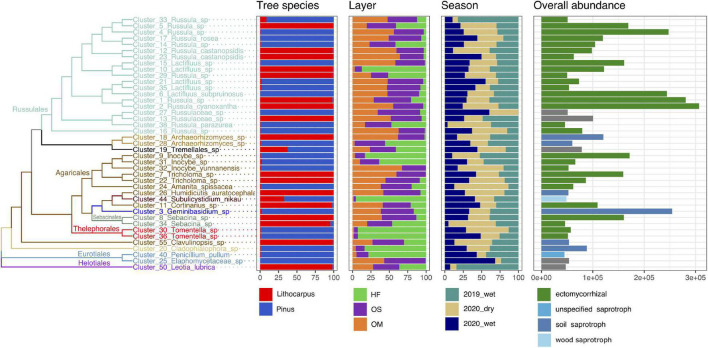
Distribution, abundance, and ecology of the most abundant soil fungal OTUs among forest associations, soil layers and seasons. The tree shows the phylogenetic relationship between the 41 most abundant fungal OTUs (accounting for 50% of the total fungal abundance). The repartition of the OTU abundance among tree species, soil layers and season of each OTU is represented as bars. HF, humic fragmented. OS, Organic soil. OM, Organo-mineral soil. 2019_wet, wet season 2019. 2020_wet, wet season 2020. 2020_dry, dry season 2020.

**FIGURE 5:**
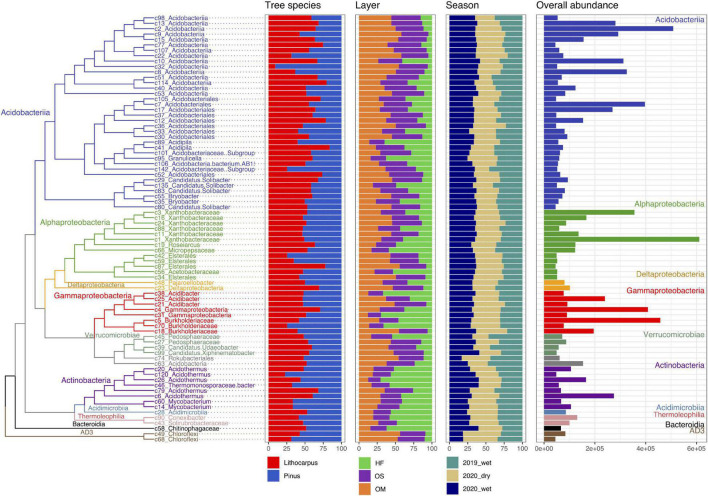
Distribution, abundance, and ecology of the most abundant soil bacterial OTUs among forest associations, soil layers and seasons. The tree shows the phylogenetic relationship between the 71 most abundant bacterial OTUs (accounting for 50% of the total fungal abundance). The repartition of the OTU abundance among tree species, soil layers and season of each OTU is represented as bars. HF, humic fragmented. OS, Organic soil. OM, Organo-mineral soil. 2019_wet, wet season 2019. 2020_wet, wet season 2020. 2020_dry, dry season 2020.

The same major lineages (Acidobacteria, Actinobacteria, and Proteobacteria for the bacterial microbiome and Agaricomycetes for the mycobiome) were observed in both stone oak and pine forests, suggesting that most of the locally distinct taxa are at the species or genera levels, rather than taxa in higher rank (Figures 4, 5 and Supplementary Figure 3). Significant variations in OTU abundance were indeed observed between *Lithocarpus-* and *Pinus-*dominated forests, despite small geographic distances among sites and similar climatic conditions. These sites are similar in terms of climate and precipitation seasonality, but strikingly differ in tree associations. Both Acidobacteria and Proteobacteria were prominent in the soil cores on both sites, but Acidobacteria were enriched in the stone oak forest (Welch’s test, *P* < 0.05, Supplementary Figure 3). Among the 20 most abundant OTUs, 10 belonged to unknown species of Acidobacteria, but *Bradyrhizobium* and *Burkholderia* species were found.

The fungal communities were dominated by members of the phylum Basidiomycota, followed by Ascomycota and Mortierellomycota. At the class level, Agaricomycetes were the most common fungi, but Archeorhizomycetes, Geminibasidiomycetes, Leotiomycetes, and Eurotiomycetes were also detected (Supplementary Figure 3). At the family level, OTUs belonging to ectomycorrhizal Russulaceae (Agaricomycetes) were the most abundant in the *Lithocarpus* and *Pinus* forests, but they were even more abundant in the old-growth forest than in the secondary woodland (Welch’s test, *P* < 0.05) (Supplementary Figure 3). OTUs belonging to the ectomycorrhizal Agaricales (e.g., *Amanita*, *Cortinarius*, *Tricholoma*) were also abundant in the stone oak forest, while Thelophorales (*Thelephora*) were more abundant in pine woodlands.

We observed substantial differences in the composition of the bacterial and fungal communities in the various soil layers and during the different sampling seasons (Supplementary Figures 3–7). The HF layer exhibited a higher proportion of Proteobacteria, Actinobacteria, Armatimonadetes, and Bacteroidetes than the OS/OM layers, while the relative abundance of Acidobacteria and several other minor phyla was lower in the HF layer (Supplementary Figures 3, 4). For the fungal community, the proportion of Agaricomycetes was slightly higher in the OS/OM layers by comparison to the HF horizon (Supplementary Figures 3, 5). Of note, Proteobacteria (Supplementary Figures 3, 6) and Agaricomycetes (Supplementary Figures 3, 7) were more abundant during the dry season compared to the wet season.

We identified specific- and enriched-OTUs in either the *Lithocarpus* or *Pinus* forests. Specific OTUs were seldom. On the other hand, the *Lithocarpus* microbiome comprised a higher number of enriched OTUs (3205 bacterial and 1284 fungal OTUs, respectively) compared to the *Pinus* forests (2769 bacterial and 1072 fungal OTUs, respectively), indicating that both bacterial and fungal communities comprised the same taxa, but in strikingly different proportions. Notably, we found that enriched OTUs affiliated to Acidobacteria and Proteobacteria were more abundant in the *Lithocarpus* forest, while enriched OTUs belonging to Actinobacteria were more abundant in the *Pinus* soil cores (Fisher’s exact test, *P* < 0.05, Supplementary Table 4).

We also found that the HF soil layer contained a higher number of specific- and enriched-OTUs by comparison to the OS/OM layers. The soil cores collected during the wet season in 2019 contained a higher proportion of specific- and enriched bacterial OTUs, whereas the number of specific- and enriched fungal OTUs were slightly higher during the wet season in 2019 and the dry 2020 season. Of note, the proportion of OTUs varying with seasonality was much higher than those affected by the soil layers or forest types.

### Dominant Taxa

**FIGURE 6:**
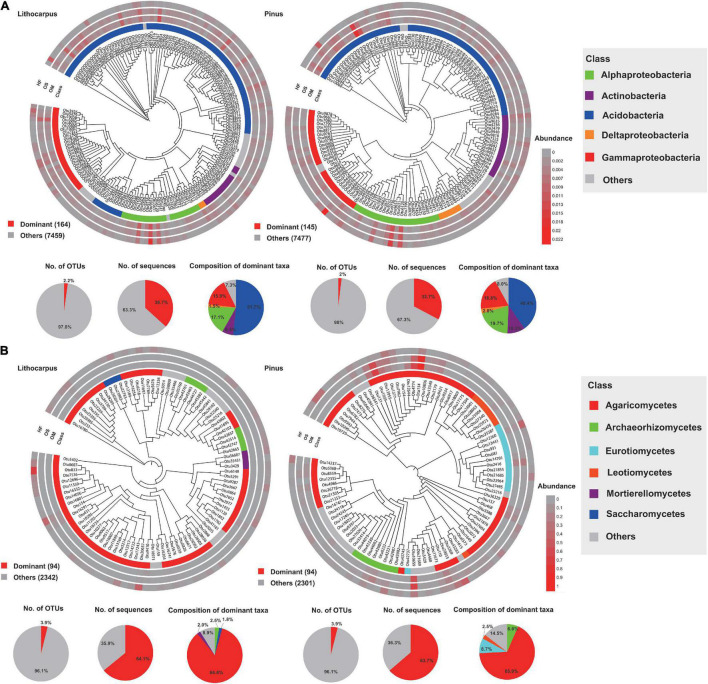
Phylogenetic tree, taxonomic composition and distribution patterns of dominant taxa. **(A)** Bacterial phyla and **(B)** fungal families in each forest association. The dominant taxa were defined as OTUs present in more than 80 and 50% of all samples and with an average relative abundance ≥ 0.1%.

We identified the dominant OTUs (present in at least 80% (bacteria) and 50% (fungi) of the samples and with a relative abundance > 0.1%) and the biomarker taxa for each forest associations (Figure 6). In the bacterial community, only 164 and 145 dominant OTUs (2.2 and 2% of the total community) were identified in the *Lithocarpus* and *Pinus* forests, while 94 and 94 dominant OTUs (3.9 and 3.9% of the total community) were identified in the *Lithocarpus* and *Pinus* forest mycobiome. They mainly belonged to Acidobacteria and they accounted for 36.7%, and 32.7% of the total sequences from each forest association, respectively. In the fungal community, they mainly belonged to Agaricomycetes, which accounted for 64.1%, and 63.7% of the total sequences in the two forests, respectively (Figure 6). In total, there were 76 bacterial and 5 fungal dominant OTUs shared by the *Lithocarpus* and *Pinus* forests. The analysis of LDA effect size (LEfSe) indicated that unknown species in the Acidobacteria and Chloroflexi were the most significant bacterial biomarkers and *Russula* (Russulaceae) and *Lactifluus* (Russulaceae) were fungal biomarkers for *Lithocarpus* and *Pinus* sites, respectively (Supplementary Figure 8).

**FIGURE 7:**
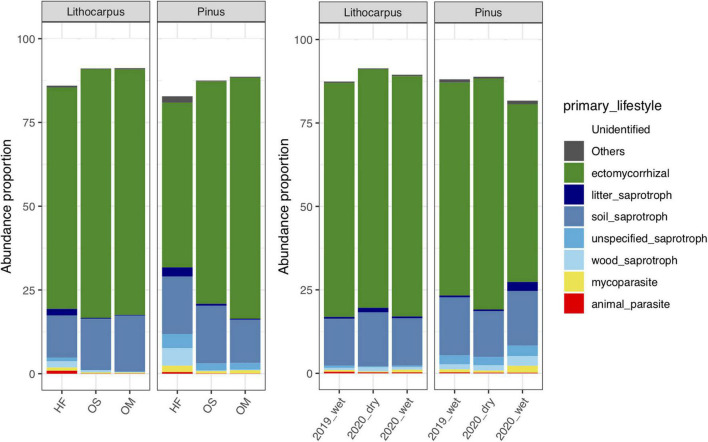
Distribution of OTUs amongst functional guilds (e.g., ectomycorrhizal symbionts, saprotrophs, pathogens, endophytes) in the soil layers or during the different seasons.

The fungal guilds were massively dominated by ectomycorrhizal taxa, representing > 50% of the OTUs in the various soil layers and forest associations during both the wet and dry seasons (Figure 7). Although ectomycorrhizal Russulaceae were prominent in soil mycobiomes of both the primary and secondary forests, different closely related taxa within this family were predominant in either the stone oak or pine forests or in the various soil layers (Figure 4). For example, Cluster_1_*Russula* cf. *subpallidirosea*, Cluster_2_*Russula*_*cyanoxantha*, Cluster_5_*Russula*_sp, Cluster_12_*Russula*_*castanopsidis/*Cluster_23 *Russula*_ *castanopsidis* and Cluster_10_*Lactifluus*_sp were amongst the most abundant OTUs of *Lithocarpus*-sampled soil cores and were seldomly detected in the pine woodland. On the other hand, Cluster_6_*Lactifluus*_*subpruinosus* and Cluster_4_*Russula*_sp were within the most abundant in pine-related soil cores and not detected in stone oak forest. Of note, the heat-resistant and xerotolerant basidiomycete Cluster_3_*Geminibasidium*_sp was only abundant in the OM/OS layers from the *Pinus* plantation soil samples, but during both the dry and wet seasons. Among the most abundant fungal OTUs, only the population of Cluster_38_*Russula*_*parazurea*, Cluster_34_*Sebacina*_sp and Cluster_24_*Amanita_spissacea* was clearly most abundant at the end of the dry season. The ITS1 sequence of fruiting bodies collected on the sites matched the sequences found in the soil DNA.

### Fungal Diversity in Roots

**FIGURE 8:**
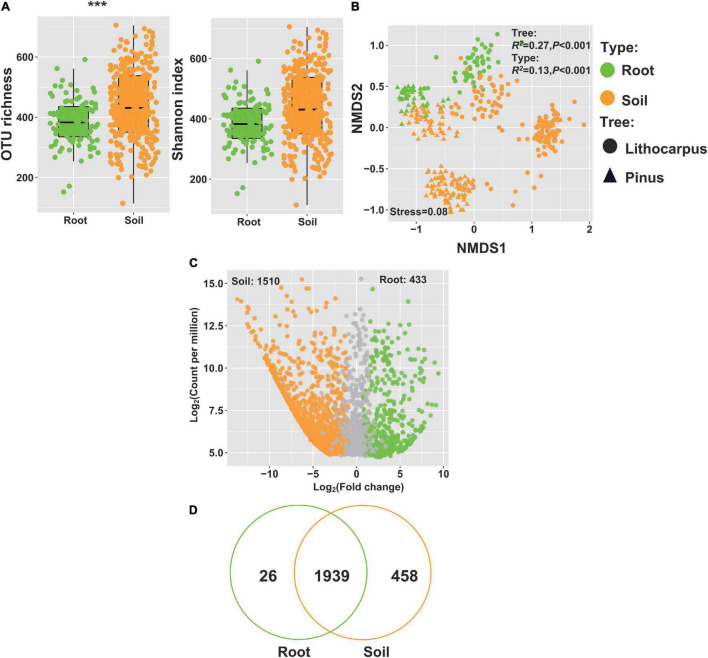
Distribution of fungal OTUs in roots and soils of the *Lithocarpus* and *Pinus* forests. **(A)** Diversity and richness of the fungal communities in root and soil samples. **(B)** NMDS ordination based on weighted UniFrac distances matrices of fungal community for roots and soil samples. **(C)** Volcano plot showing the relative abundance (fold change) of OTUs in soil (orange dots) and roots (green dots). **(D)** Venn diagram showing OTUs found either in soil or roots or both.

In both stone oak and pine forests, ectomycorrhizal fungi, such as *Russula*, *Lactarius*, *Lactifluus*, *Tricholoma, Inocybe*, or *Cortinarius*, were dominant in the soil mycobiome, irrespective of the soil layers and season (Figure 4). Thus, we surveyed the fungal composition of roots sampled underneath stone oaks or pines. The alpha-diversity was higher in *Pinus* roots than in *Lithocarpus* roots (Supplementary Figure 9A). As shown by the ordination analysis, the root fungal community, comprising > 320 OTUs, was significantly different between the tree species (*R*^2^ = 50%, *P* < 0.001; Supplementary Figure 9) and the sampling season had also a significant influence on the fungal composition of the roots (*R*^2^ = 10%, *P* < 0.001, Figure 8B). As expected, the alpha-diversity of the root mycobiome was lower than the soil mycobiome (Figure 8A) and their composition varied significantly with the forest associations (*P* < 0.001, Figure 8B). The Agaricomycetes represented > 75% of the detected OTUs associated to *Lithocarpus* and 50% in pine roots. Russulaceae comprised the largest set of OTUs in *Lithocarpus* roots, while Thelophoraceae were the most prominent in *Pinus* roots (Supplementary Figure 9D). As expected, most of the OTUs detected in roots were also found in the soil community (Figure 8D), although up to 433 OTUs were enriched in roots and 26 appeared to be specific to roots (Figure 8C).

## Discussion

### Microbial Diversity in the Old-Growth Forest and Pine Woodland

On a global scale, microbial richness is mainly driven by the relative distribution and richness of host plants, soil pH, mean annual temperature and mean annual precipitations (Wardle et al., 2004; Tedersoo et al., 2014). In Ailaoshan forests, we found that the bacterial and fungal communities are strongly shaped by the forest type (primary vs. secondary associations), confirming the importance of tree-microbe interactions in shaping tropical forest structure (Geml et al., 2017; Adamo et al., 2021; Wang et al., 2022). Seasonality and soil features also influenced microbial richness and composition. Microbial richness appeared to be significantly higher in the pine woodlands, corresponding to a late-successional (>60 years) stage, by comparison to the old-growth primary stone oak forest. It is widely recognized that forest age impacts microbial diversity (Norden and Paltto, 2001; Chaverri and Vilchez, 2006; Nie et al., 2012). Several surveys of forest soils have shown that fungal diversity was higher in the younger forests compared to the older forests (Norden and Paltto, 2001; Chaverri and Vilchez, 2006), supporting the intermediate disturbance hypothesis. On the other hand, Nie et al. (2012) found that the difference in soil bacterial community was limited between natural old-growth *Pinus massaniana* forests and nearby secondary plantations in subtropical China. However, they also showed that the natural forest hosted a higher diversity of fungi in comparison with the young planted forest. These findings contrast with previous reports showing that replacement of deciduous temperate forests by conifer plantations led to a lower diversity of soil bacterial and fungal community composition (Buée et al., 2009; Kutszegi et al., 2015).

Our findings also revealed significant changes in the microbial beta-diversity between the old-growth *Lithocarpus* forest and the successional woodlands of the native Yunnan pines. However, despite substantial differences in the composition of the bacterial and fungal communities among *Lithocarpus* and *Pinus* forests, several of the prominent lineages/OTUs occurred in both forest types, indicating no limit in the microbial dissemination between the primary and nearby secondary forests. On the other hand, the microbial communities inhabiting the old-growth forest may have flourished on the new pine hosts following forest replacement because they survived in the spore bank. This being said, the relative abundance of OTU populations was strikingly different between the stone oak and pine forests, with closely related taxa often occupying either the primary or the secondary forests sites. These findings suggest that most soil bacteria and fungi are widely distributed across the Ailaoshan region and their occurrence is constrained by contemporary environmental factors. Most shared species displayed niche preferences to either the stone oak-dominated forest or the pine woodlands supporting this contention.

Although the ecological/biological mechanisms responsible for the observed diversity patterns remain unclear, the greater microbial composition among plots in the secondary pine woodlands than in primary stone oak forests may be explained partly by higher stochasticity in colonization following disturbance (including human activities). As a consequence, microbial communities may have experienced greater variations of edaphic variables. The slightly lower alpha-diversity of the mycobiome in the primary forest stands suggests more similar microbial communities, likely because of environmental filtering under relatively stable conditions over centuries, when compared to the secondary pine woodlands. It has been suggested that increased environmental filtering and competition for more limited resources may lead to compositional convergence in primary forests. This is also supported by the highest number of biomarker taxa, specific- or enriched OTUs.

### Soil Characteristics and Seasonality Shape the Microbial Communities

The role of soil attributes as one of the main drivers of the microbial community assembly in forests has been widely recognized in boreal and temperate forest ecosystems (Lindahl et al., 2007; Wardle, 2011; Eilers et al., 2012; Taylor et al., 2014; Uroz et al., 2016). Species distribution within bacterial community is strongly influenced by soil pH, whereas variability in soil C and N contents has a profound effect on fungal community (Lauber et al., 2008; Bahram et al., 2018). In the Ailaoshan forests, we also found that the microbial communities were structured at the stand scale according to soil layers; the diversity and richness of bacterial and fungal communities were higher in the forest floor (i.e., the humic soil layer, HF) than in the organic (OS) and organo-mineral (OM) layers. As previously found by terminal restriction fragment length polymorphism (T-RFLP) analysis of 16S rRNA genes (Chan et al., 2006), Acidobacteria and Proteobacteria were the prominent soil bacterial taxa in the old-growth stone oak forest in Ailaoshan. It is widely known that soil acidity is one of the most important selection factors determining growth of Acidobacteria in most terrestrial ecosystems. Proteobacteria is also frequently found in the acidic conditions of forest soils, where they are often predominant (Nie et al., 2012; Xiong et al., 2021b). On the other hand, the fungal diversity and richness were the highest in HF and the lowest in OS soil layer.

### The Soil Mycobiome Is Dominated by Ectomycorrhizal Species

In subtropical forests, ectomycorrhizal and arbuscular mycorrhizal plants often co-occur at comparable abundances (Wu et al., 2013; Toju et al., 2014). In contrast, the evergreen sclerophyllous broad-leaved subtropical forest biome and the secondary woodlands in Ailaoshan mostly comprise ectomycorrhizal trees. The symbiotic fungi associated to stone oaks and pines were ectomycorrhizal species within the genera *Russula, Lactifluus, Tricholoma*, and *Inocybe*. Ericoid mycorrhizal fungi were not found despite the fact that ericaceous dwarf-shrubs, such as *Rhododendron* spp., are understory plants in both the old-growth forest and pine woodlands. Saprotrophic fungi were present in low abundance, although we found several OTUs of Geminibasidiomycetous yeasts and “sugar fungi” in Mortierellaceae.

As shown previously, the substantial change in the aboveground litter quality is a critical driver of the composition of fungal communities in boreal and temperate forests (Lindahl et al., 2007; Taylor et al., 2014). In Ailaoshan forests, the distribution of hundreds of OTUs was also strikingly different between soil layers. Ectomycorrhizal Russulaceae were dominating the mycobiome (>38%); 20 of the 40 most abundant taxa belong to Russulaceae. Several prominent *Russula* phylogroups matched known species, such as *Russula* cf. *foetens* and *Russula* cf. *subpallidirosea*, but most OTUs were unique, previously unsequenced, and with or without known close relatives. These taxa of unknown identity may or may not represent newly discovered species, awaiting a formal description. *Russula* and *Lactifluus* species showed strong habitat preference not only to one of the two major forest types, i.e., *Lithocarpus* vs. *Pinus*, but often to soil layers as well. Some were mainly detected in the OM and OS soil horizons, but others were more abundant in the HF layer. These differential spatial patterns suggest clear differences in habitat preference even within functional groups and clades (Chan et al., 2006; Lauber et al., 2008; Taylor et al., 2014; Uroz et al., 2016), suggesting that deterministic niche partitioning in community assembly is a major driver. We speculate that this shift in the fungal composition is likely related to the striking changes in organic matter (and related carbon) and organic N content in soil layers.

Based on their sheer abundance and wide distribution in both old-growth and secondary forests, Russulaceae species presumably have great ecological importance as mycorrhizal partners of trees in subtropical ecosystems, as found in *Pinus massoniana* plantations in the Sichuan Province, China (Li et al., 2021) and in a subtropical secondary forest with evergreen Fagaceae species (Toju et al., 2014). Russulaceae and Thelephoraceae were also the most abundant families in soils of a *Castanopsis*-dominated subtropical forest located in the Gutianshan National Nature Reserve in Zhejiang in Southeastern China (Wu et al., 2013), suggesting a global pattern of distribution in subtropical forests for these two ectomycorrhizal fungal families. *Tomentella/Thelephora*, *Cortinarius* and *Russula* species were the most prominent OTUs in the dipterocarp and montane forests of Mount Kinabalu in Borneo (Geml et al., 2017). The prominence of *Russula* and *Lactarius/Lactifluus* symbionts, characterized by medium-smooth, short distance or contact extramatrical mycelial exploration types, respectively, may be related to a fast turnover of decomposing SOM leading to high pools of labile N (Hobbie and Agerer, 2010).

Precipitation is a well-known abiotic variable with a strong positive influence on fungal diversity on a global scale (Tedersoo et al., 2014; Wang et al., 2022). As expected, we found that within each soil layer, the composition of the bacterial and fungal soil microbiomes varied with the season and its richness was the highest during the high precipitation monsoon period. This is consistent with previous studies carried out in temperate and tropical forests (Žifèáková et al., 2016; Adamo et al., 2021; Oita et al., 2021; Wang et al., 2022). Climate and seasonality are key drivers of soil microbial biodiversity. Understanding the impacts of shifts in seasonality on the functions expressed by tree-associated microbial communities is needed for getting a better understanding of the impact of climate changes on subtropical forest ecosystems.

The current survey of soil microbial communities in response to forest replacement from primary old-growth broadleaf forest to native pine woodlands provides a comprehensive overview of compositional diversity and their potential functional changes upon changes in forest management. Our sampling involved both soils and roots and was conducted at multiple sites of each forest type as well as across time periods that captured the wet and dry seasons. Although previous studies assessed the extent to which forests dominated by different plant communities result in changes in microbial communities in subtropical montane forests in Asia (Miyamoto et al., 2015; Geml et al., 2017; Wang et al., 2022), we provide here the first insight into the composition of both bacterial and fungal communities from the evergreen sclerophyllous broad-leaved subtropical forest biome, the most extensive subgroup in the subtropical biome which historically covered most of southern China. Our results showed that the shift from primary to secondary forests mediated alterations in below-ground microbial communities, altering the composition and diversity of bacteria and fungi, but did not lead to a loss of biodiversity, i.e., species richness. However, it remains to determined how critical functions expressed by these microbial communities are altered by forest replacement. The present study is also providing fundamental information on microbial diversity in contrasting land types, secondary woodlands vs. old-growth forests, which may be relevant to policy and management as the international community is increasingly focusing on land conversion and tree-plantings.

## Data Availability Statement

Raw sequences have been submitted to the Sequence Read Archive under accession number: PRJNA782391.

## Author Contributions

FM coordinated the project. FM, MB, GW, YD, and ZY designed the experiments. QZ and AL analyzed the data with the help of MB and FM. QZ, AL, and FM wrote the manuscript with input from the other authors. All authors were involved in the field sampling.

## Conflict of Interest

The authors declare that the research was conducted in the absence of any commercial or financial relationships that could be construed as a potential conflict of interest.

## Publisher’s Note

All claims expressed in this article are solely those of the authors and do not necessarily represent those of their affiliated organizations, or those of the publisher, the editors and the reviewers. Any product that may be evaluated in this article, or claim that may be made by its manufacturer, is not guaranteed or endorsed by the publisher.

## Footnotes

1
www.globalforestwatch.org

2
https://deims.org/13f23bec-9cc6-4b31-9032-fcbe850b448c

3
https://cloud.oebiotech.cn

4
https://itol.embl.de/

5
http://huttenhower.sph.harvard.edu/galaxy/

## Supplementary Material

Supplementary Figure 1 **(A)** Map of the sampling Ailaoshan sites with the old-growth stone oak (*Lithocarpus*) forest (top right yellow arrow) and secondary Yunnan pine plantation (bottom left yellow arrow). **(B)** Site maps showing the location of the three clusters of four trees for each sampling site.

Supplementary Figure 2 Physicochemical properties of the soil cores collected in the primary *Lithocarpus* forest and the secondary Yunnan pine plantations in the organic soil layer (OS) and organo-mineral soil (OM). The asterisks indicate the properties significantly different between *Lithocarpus* and *Pinus* samples (**P* < 0.05; ^**^*P* < 0.01; ^***^*P* < 0.001; Wilcoxon test). CEC, cation exchange capacity.

Supplementary Figure 3 The relative abundance of the most prominent bacterial phyla **(A)**, fungal classes **(B),** and fungal families **(C)** in the *Lithocarpus* or *Pinus* soil cores, in the different soil layers **(D,E)** and various sampling seasons **(F,G)**. Differential abundance of bacterial **(H)** and fungal **(I)** OTUs in the *Lithocarpus* and *Pinus* forests. Welch’s tests followed by Benjamini–Hochberg FDR corrections were performed between different forest associations. The phyla, classes and families with less than 0.05 or 0.5% of the average relative abundance are grouped into “Others.”

Supplementary Figure 4 The relative abundance of dominant bacterial phyla among soil layers. Different letters above the boxes indicate a significant difference. The phylum with less than 0.05% of the average relative abundance are grouped into “Others.”

Supplementary Figure 5 The relative abundance of dominant fungal classes among soil layers. Different letters above the boxes indicate a significant difference. The classes with less than 0.05% of the average relative abundance are grouped into “Others.”

Supplementary Figure 6 The relative abundance of dominant bacterial phylum over the sampling seasons. Different letters above the boxes indicate a significant difference. The phyla with less than 0.05% of the average relative abundance are grouped into “Others.”

Supplementary Figure 7 The relative abundance of dominant fungal class over sampling seasons. Different letters above the boxes indicate a significant difference. The classes with less than 0.05% of the average relative abundance are grouped into “Others.”

Supplementary Figure 8 The specific, shared and biomarker taxa of the bacterial **(A)** and fungal **(B)** microbiomes in soil cores of the *Lithocarpus* and *Pinus* forests. The top 10 most specific biomarker taxa are shown for bacterial **(C)** and fungal microbiome **(D)**. LEfSe was used to identify biomarker taxa.

Supplementary Figure 9 Distribution of fungal OTUs in roots of the *Lithocarpus* and *Pinus* forests. **(A)** OTU richness and alpha-diversity in *Lithocarpus* and *Pinus* forests. **(B)** NMDS ordination based on weighted UniFrac distances matrices of fungal community for root samples. **(C)** Distribution of OTUs among fungal families in the *Lithocarpus* and *Pinus* roots samples. **(D)** Distribution of root OTUs among fungal families.

Supplementary Table 1 Effects of multiple biotic and abiotic factors on the microbiome assembly of the *Lithocarpus* and *Pinus* forests.

Supplementary Table 2 Effects of the soil layers and seasons on bacterial and fungal alpha diversity based on linear mixed model (LMM). Significance was assessed using Satterthwaite’s method.

Supplementary Table 3 Correlation coefficients between alpha-diversity and environmental variables. (**P* < 0.05; ^**^*P* < 0.01).

Supplementary Table 4 Bacterial and fungal OTUs with enriched distribution in the soil cores of the *Lithocarpus* and *Pinus* forests.
