# Supplementary figures and images for: Ecological Drivers of the Soil Microbial Diversity and Composition in Primary Old-Growth Forest and Secondary Woodland in a Subtropical Evergreen Broad-Leaved Forest Biome in the Ailao Mountains, China
Source: Front Microbiol. 2022 Jun 13;13:908257. doi: 10.3389/fmicb.2022.908257 (PMC9234548; doi:10.3389/fmicb.2022.908257)

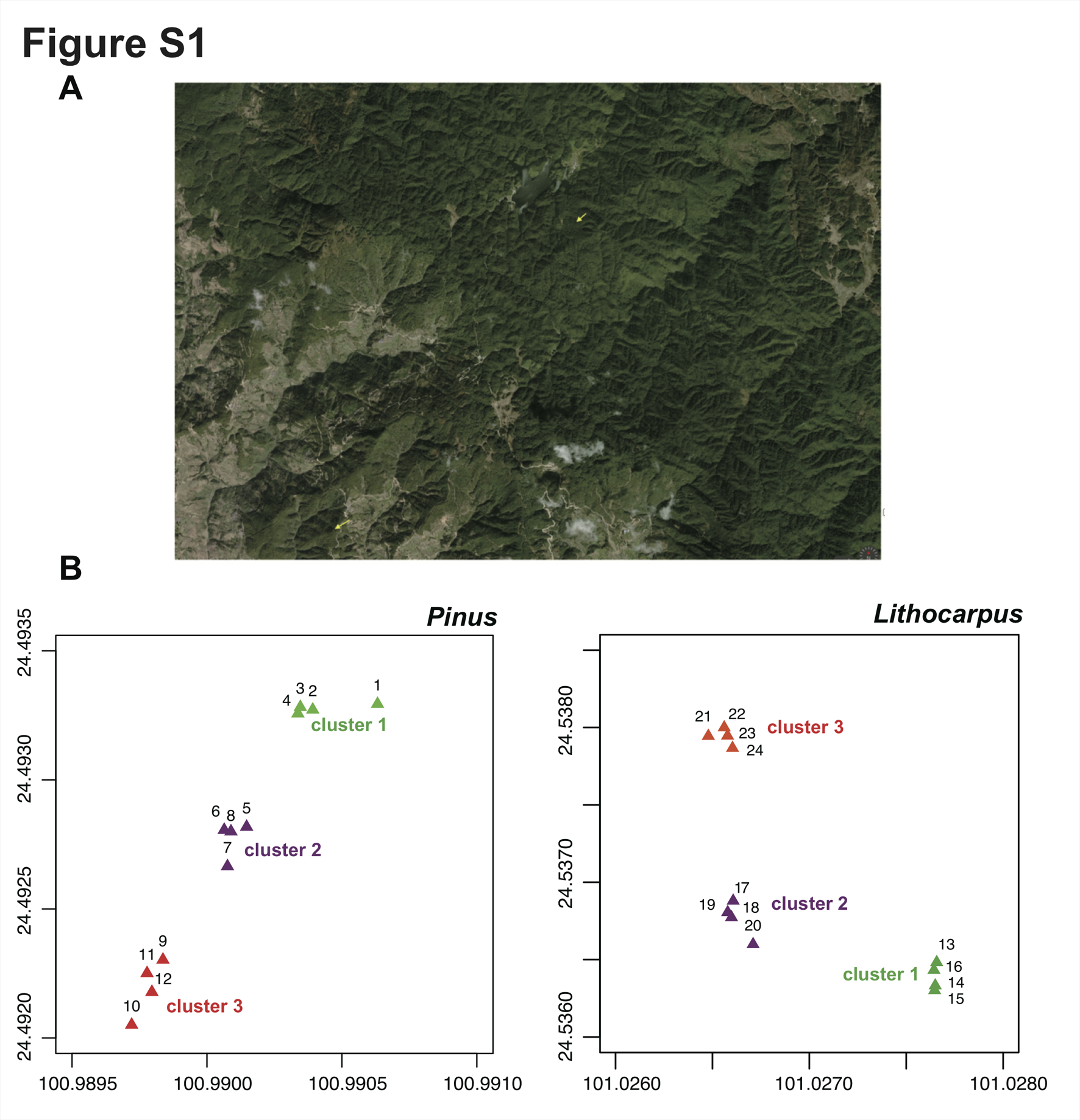

Supplement: Supplementary Figure 1 — (A) Map of the sampling Ailaoshan sites with the old-growth stone oak (Lithocarpus) forest (top right yellow arrow) and secondary Yunnan pine plantation (bottom left yellow arrow). (B) Site maps showing the location of the three clusters of four trees for each sampling site. [file Image_1.TIFF]

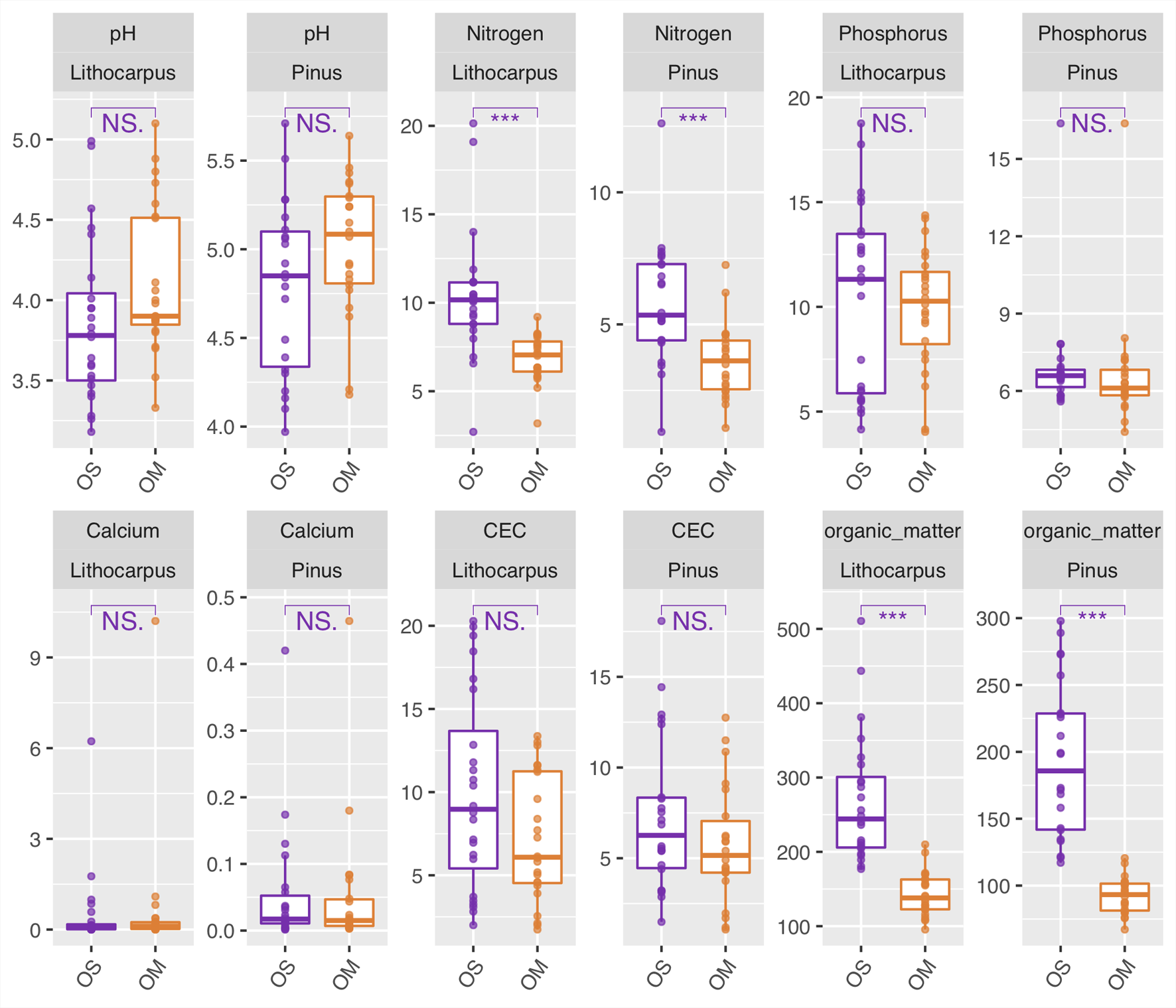

Supplement: Supplementary Figure 2 — Physicochemical properties of the soil cores collected in the primary Lithocarpus forest and the secondary Yunnan pine plantations in the organic soil layer (OS) and organo-mineral soil (OM). The asterisks indicate the properties significantly different between Lithocarpus and Pinus samples (*P < 0.05; **P < 0.01; ***P < 0.001; Wilcoxon test). CEC, cation exchange capacity. [file Image_2.TIFF]

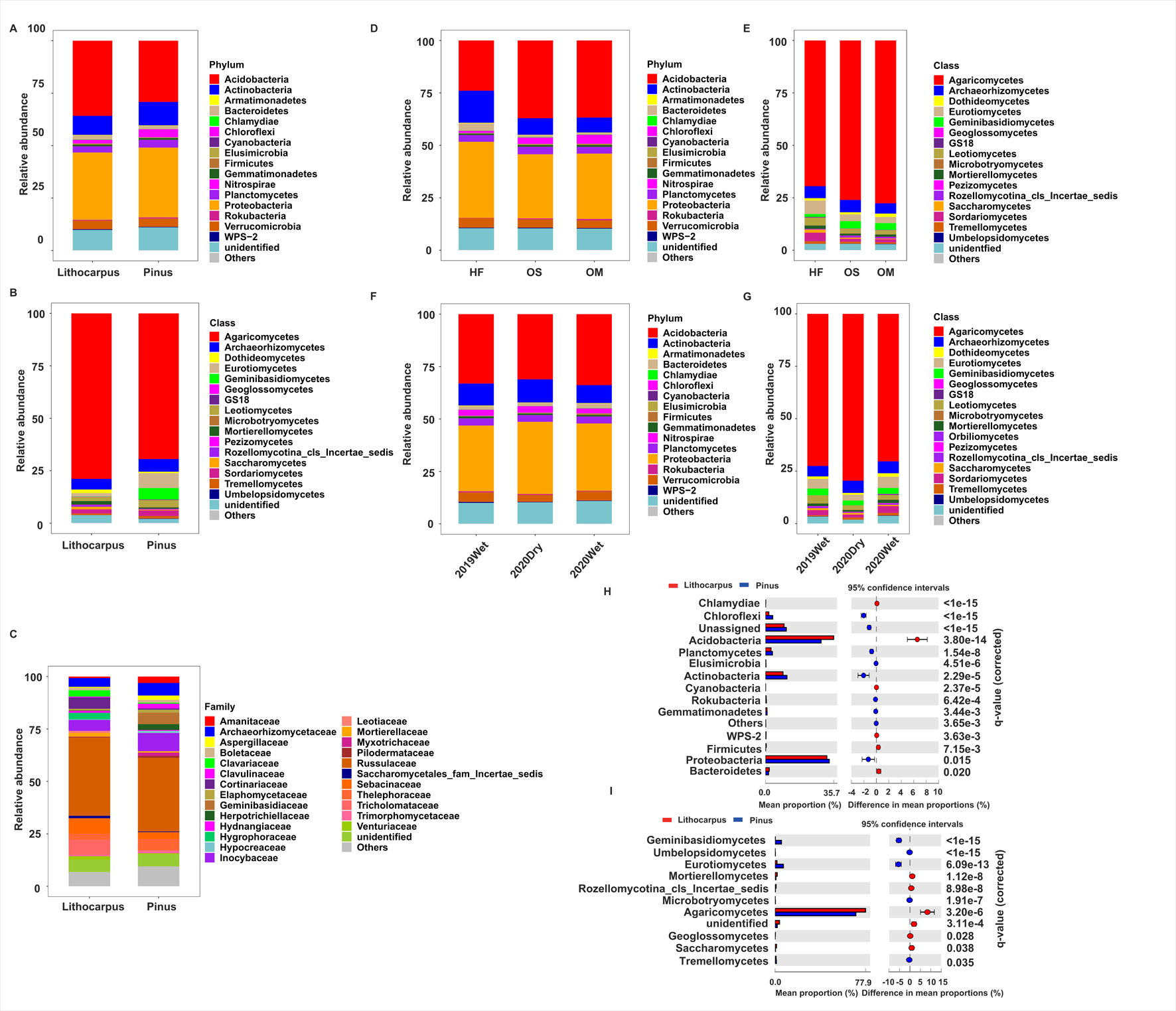

Supplement: Supplementary Figure 3 — The relative abundance of the most prominent bacterial phyla (A), fungal classes (B), and fungal families (C) in the Lithocarpus or Pinus soil cores, in the different soil layers (D,E) and various sampling seasons (F,G). Differential abundance of bacterial (H) and fungal (I) OTUs in the Lithocarpus and Pinus forests. Welch’s tests followed by Benjamini–Hochberg FDR corrections were performed between different forest associations. The phyla, classes and families with less than 0.05 or 0.5% of the average relative abundance are grouped into “Others.” [file Image_3.TIF]

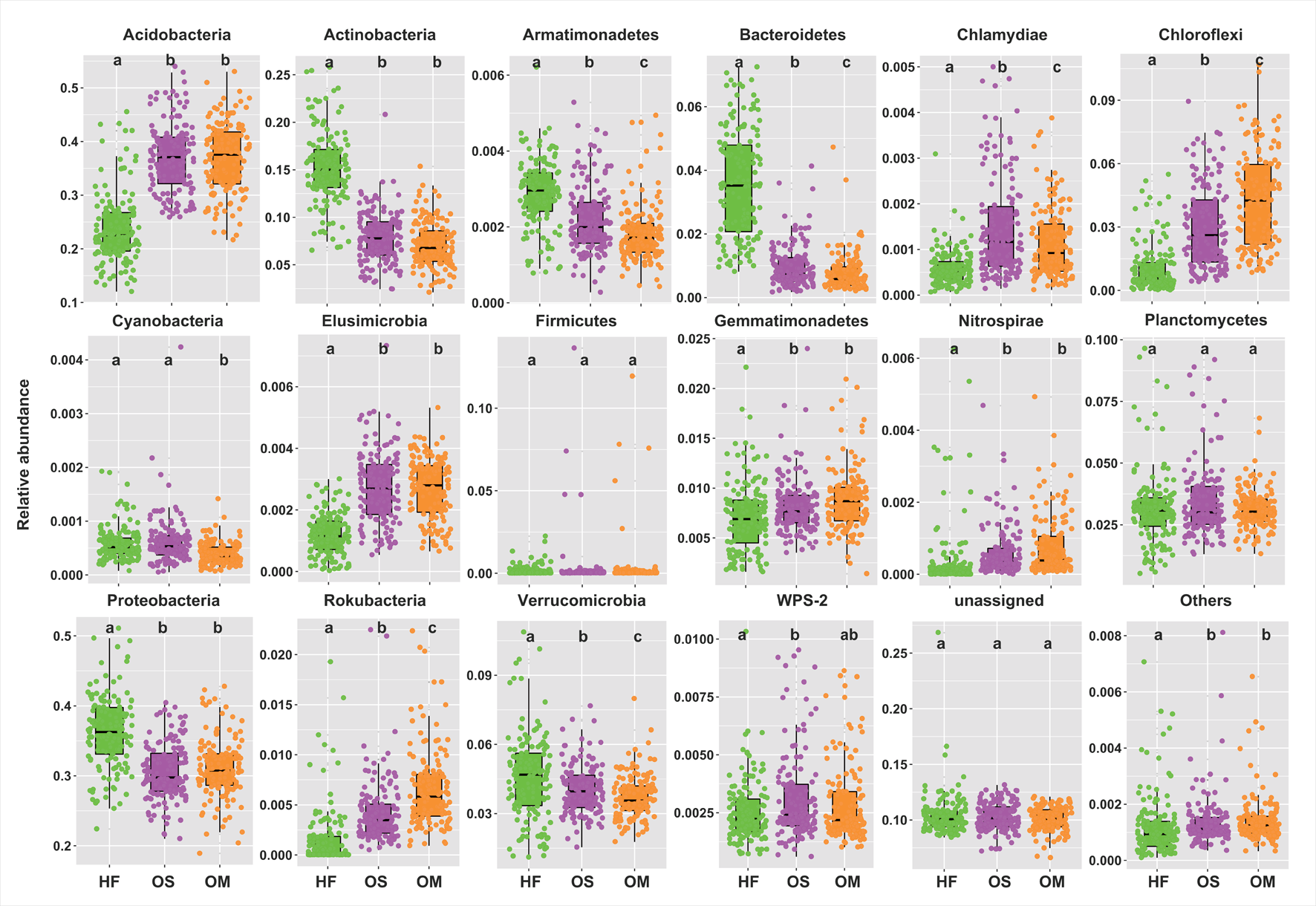

Supplement: Supplementary Figure 4 — The relative abundance of dominant bacterial phyla among soil layers. Different letters above the boxes indicate a significant difference. The phylum with less than 0.05% of the average relative abundance are grouped into “Others.” [file Image_4.TIF]

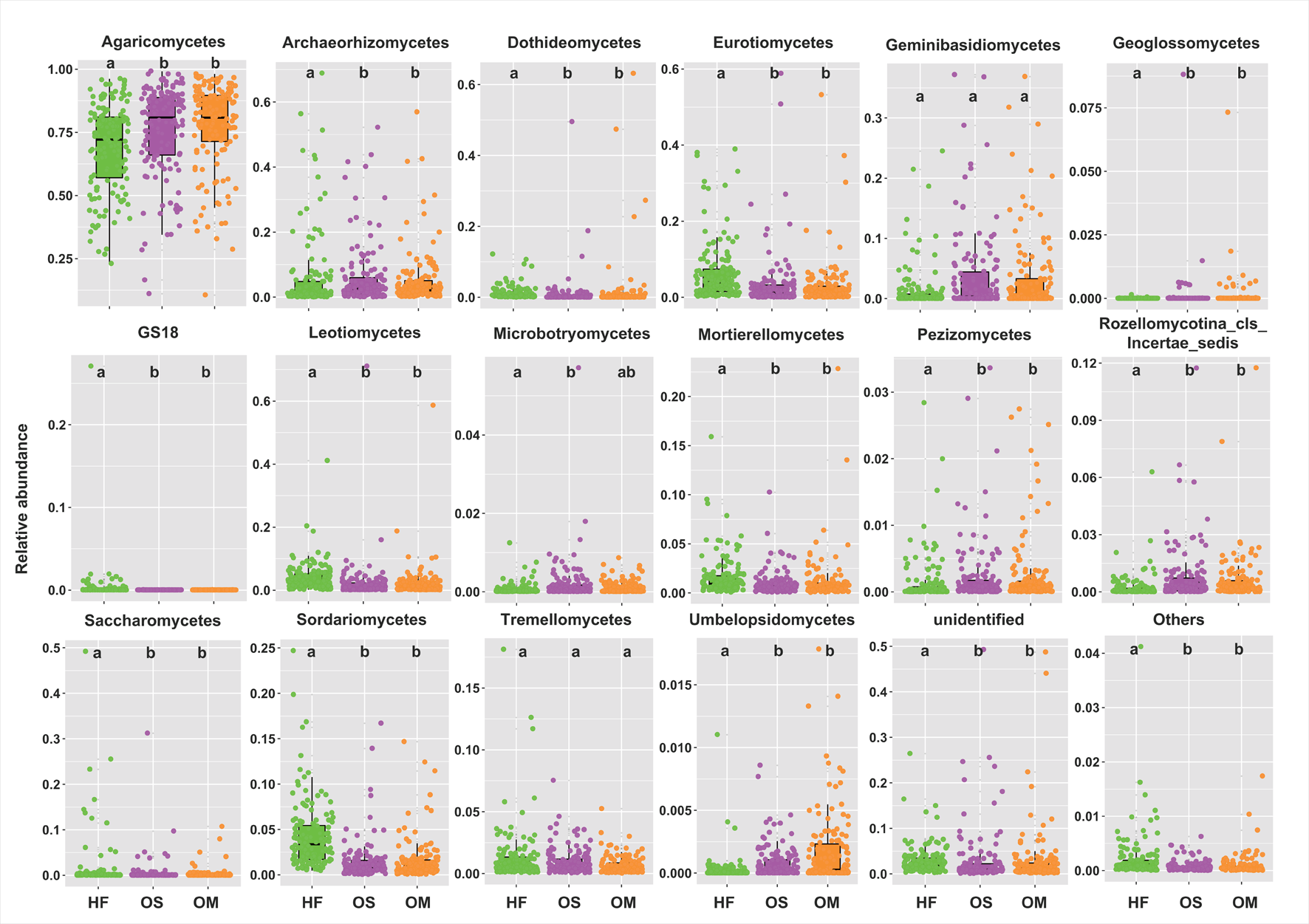

Supplement: Supplementary Figure 5 — The relative abundance of dominant fungal classes among soil layers. Different letters above the boxes indicate a significant difference. The classes with less than 0.05% of the average relative abundance are grouped into “Others.” [file Image_5.TIF]

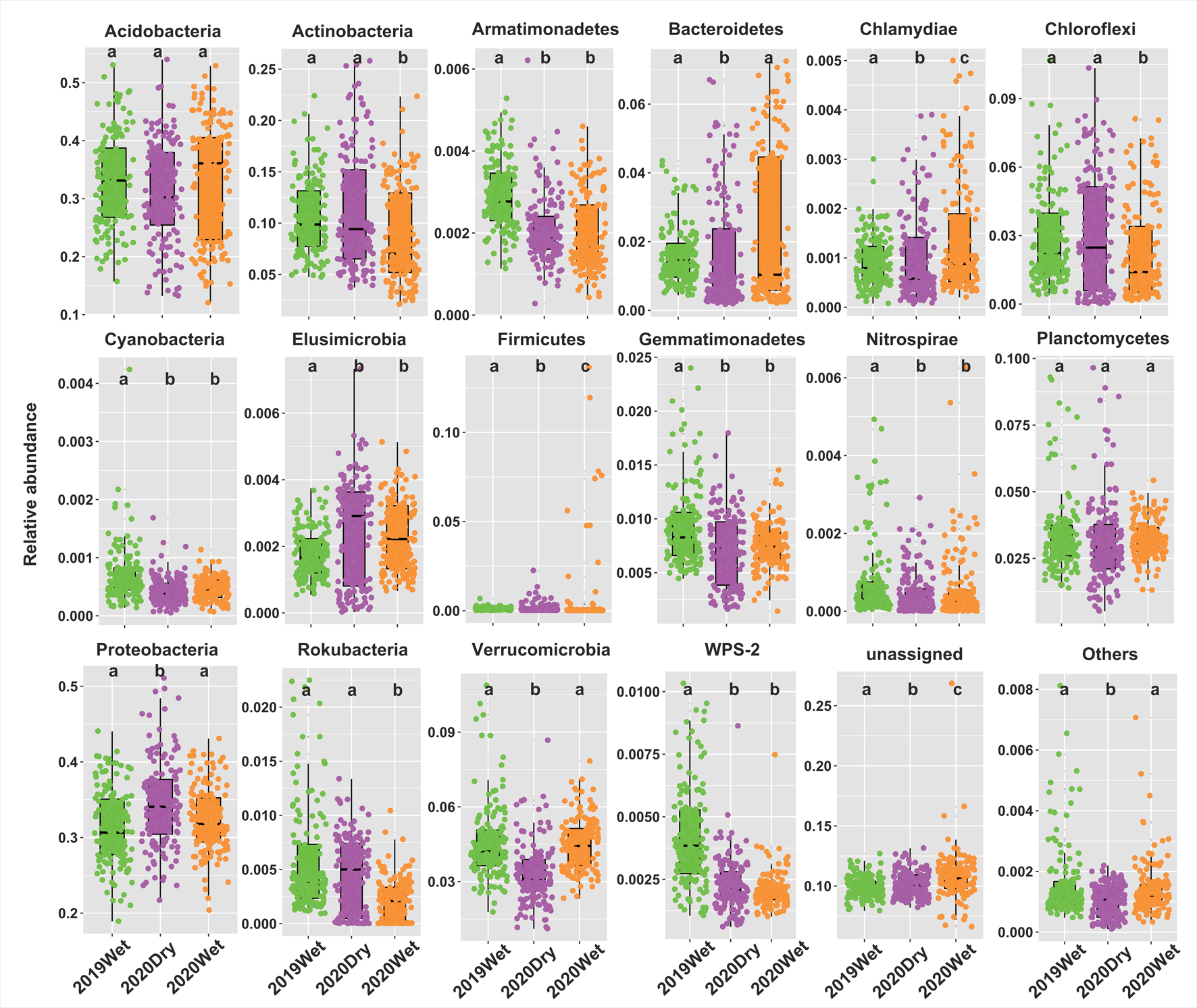

Supplement: Supplementary Figure 6 — The relative abundance of dominant bacterial phylum over the sampling seasons. Different letters above the boxes indicate a significant difference. The phyla with less than 0.05% of the average relative abundance are grouped into “Others.” [file Image_6.TIF]

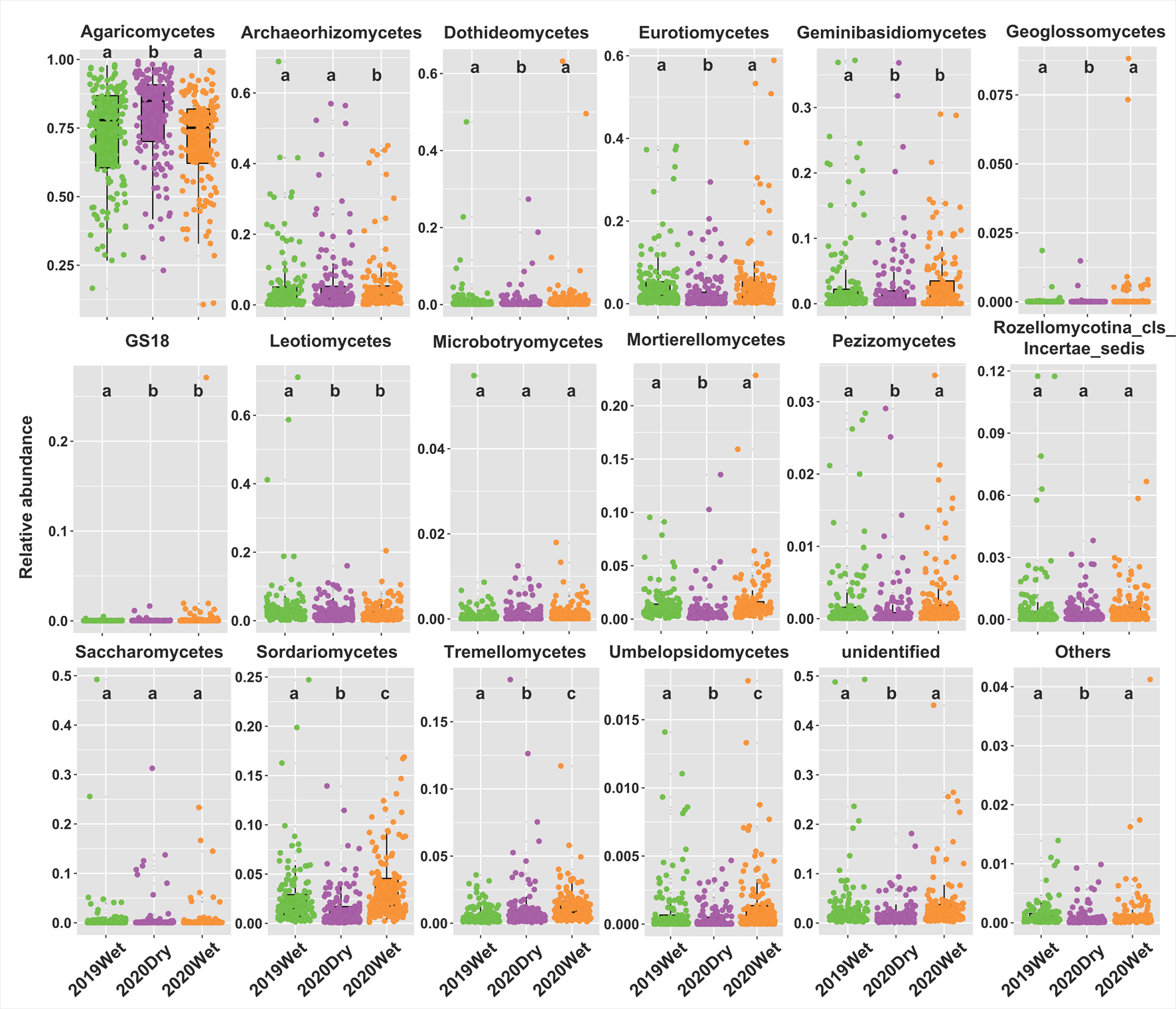

Supplement: Supplementary Figure 7 — The relative abundance of dominant fungal class over sampling seasons. Different letters above the boxes indicate a significant difference. The classes with less than 0.05% of the average relative abundance are grouped into “Others.” [file Image_7.TIF]

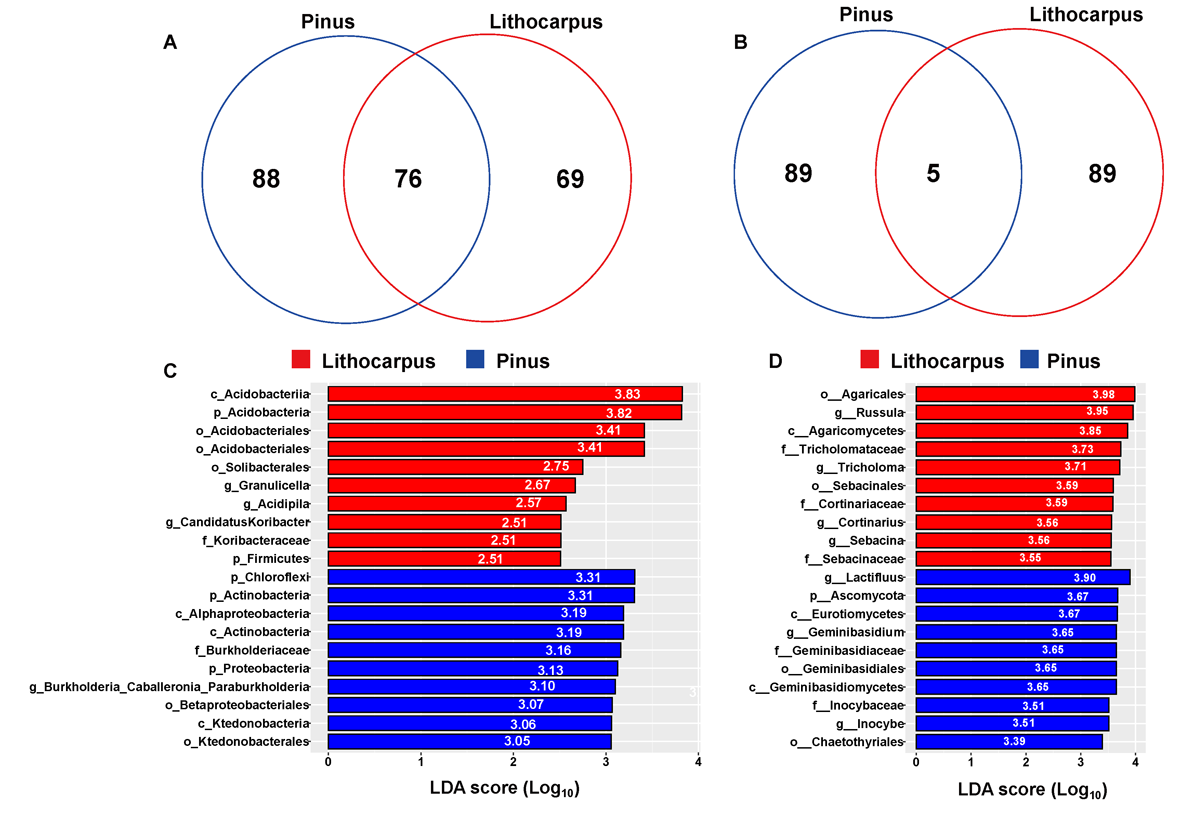

Supplement: Supplementary Figure 8 — The specific, shared and biomarker taxa of the bacterial (A) and fungal (B) microbiomes in soil cores of the Lithocarpus and Pinus forests. The top 10 most specific biomarker taxa are shown for bacterial (C) and fungal microbiome (D). LEfSe was used to identify biomarker taxa. [file Image_8.TIF]

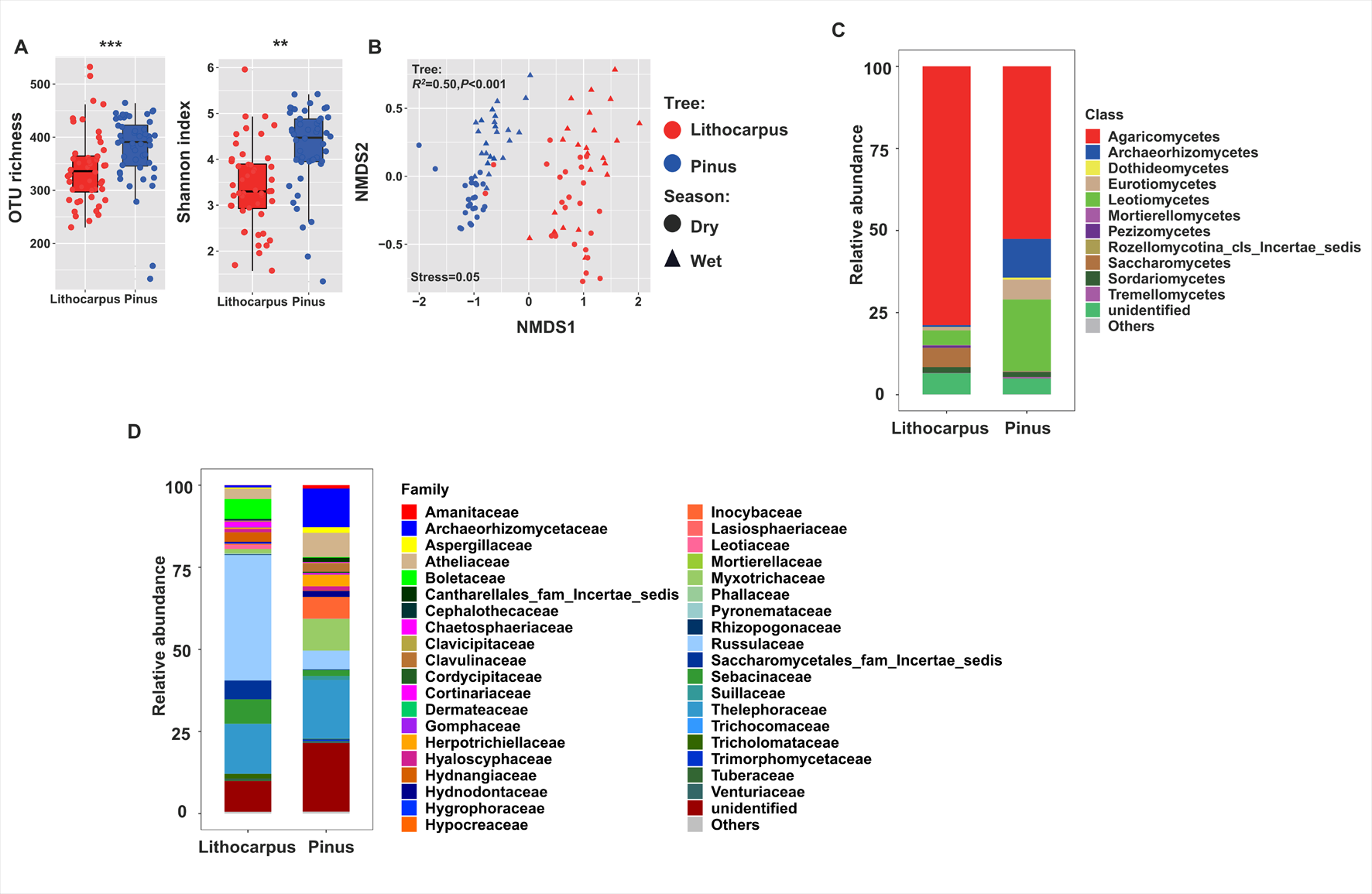

Supplement: Supplementary Figure 9 — Distribution of fungal OTUs in roots of the Lithocarpus and Pinus forests. (A) OTU richness and alpha-diversity in Lithocarpus and Pinus forests. (B) NMDS ordination based on weighted UniFrac distances matrices of fungal community for root samples. (C) Distribution of OTUs among fungal families in the Lithocarpus and Pinus roots samples. (D) Distribution of root OTUs among fungal families. [file Image_9.TIF]
